# mTOR kinase inhibitors synergize with histone deacetylase inhibitors to kill B-cell acute lymphoblastic leukemia cells

**DOI:** 10.18632/oncotarget.2992

**Published:** 2014-12-12

**Authors:** Brandon R. Beagle, Duc M. Nguyen, Sharmila Mallya, Sarah S. Tang, Mengrou Lu, Zhihong Zeng, Marina Konopleva, Thanh-Trang Vo, David A. Fruman

**Affiliations:** ^1^ Department of Molecular Biology & Biochemistry, University of California, Irvine, CA; ^2^ Department of Leukemia, University of Texas MD Anderson Cancer Center, Houston, TX; ^3^ Department of Stem Cell Transplantation and Cellular Therapy, University of Texas MD Anderson Cancer Center, Houston, TX

**Keywords:** Leukemia, B-ALL, mTOR, histone deacetylase inhibitor

## Abstract

High activity of the mechanistic target of rapamycin (mTOR) is associated with poor prognosis in pre-B-cell acute lymphoblastic leukemia (B-ALL), suggesting that inhibiting mTOR might be clinically useful. However, emerging data indicate that mTOR inhibitors are most effective when combined with other target agents. One strategy is to combine with histone deacetylase (HDAC) inhibitors, since B-ALL is often characterized by epigenetic changes that silence the expression of pro-apoptotic factors. Here we tested combinations of mTOR and pan-HDAC inhibitors on B-ALL cells, including both Philadelphia chromosome-positive (Ph+) and non-Ph cell lines. We found that mTOR kinase inhibitors (TOR-KIs) synergize with HDAC inhibitors to cause apoptosis in B-ALL cells and the effect is greater when compared to rapamycin plus HDAC inhibitors. The combination of TOR-KIs with the clinically approved HDAC inhibitor vorinostat increased apoptosis in primary pediatric B-ALL cells *in vitro*. Mechanistically, TOR-KI and HDAC inhibitor combinations increased expression of pro-death genes, including targets of the Forkhead Box O (FOXO) transcription factors, and increased sensitivity to apoptotic triggers at the mitochondria. These findings suggest that targeting epigenetic factors can unmask the cytotoxic potential of TOR-KIs towards B-ALL cells.

## INTRODUCTION

B-ALL is the most common malignancy in children and also affects adults [1, 2]. B-ALL is generally responsive to standard chemotherapy, but a subset of patients have poor prognosis. Among these high-risk cases, around 30% of adult B-ALL have the Philadelphia chromosome (Ph+); the percentage of Ph+ is much lower in pediatric B-ALL. However, a subset of “Ph-like” B-ALLs have elevated tyrosine kinase activity [3-5], raising the possibility of treatment with TKIs or agents targeting downstream components like mTOR. Elevated mTOR signaling correlates with poor prognosis in B-ALL patients [6, 7], making it a promising therapeutic target.

The serine/threonine kinase mTOR has an important role in cell growth, proliferation and survival [8, 9]. mTOR integrates signals from growth factors, nutrient availability and cell stress to produce an appropriate cellular response to growth conditions. The mTOR enzyme acts in two distinct complexes, mTORC1 and mTORC2, with different regulation and downstream substrates [8, 10]. The classical mTOR inhibitor rapamycin (RAP) and its analogs (rapalogs) bind to an allosteric site in the mTORC1 complex and reduce mTORC1 activity towards some but not all substrates [11-13]. Newer ATP-competitive mTOR kinase inhibitors (TOR-KIs) suppress phosphorylation of all mTORC1 and mTORC2 substrates [14, 15].

In cancer cells, oncogene activation leads to elevated mTOR activity, promoting survival and biosynthetic processes necessary for cell division [16-18]. There is strong interest in targeting mTOR for cancer therapy, in part based on genetic studies showing selective effects on tumor cells following mTOR inactivation [19, 20]. TOR-KIs have promising anti-tumor activity in preclinical models and several candidate compounds are in clinical trials [21-23]. Evidence is growing that TOR-KIs and rapalogs are most effective against cancer cells when used in combination with other therapies [24-28]. For example, we have reported that the tool compound PP242 and an investigational agent, MLN0128, potentiate the efficacy of tyrosine kinase inhibitors (TKIs) in B-ALL cells expressing the BCR-ABL oncoprotein [27, 28]. In a mouse BCR-ABL-dependent leukemia model, PP242 was more effective than RAP when combined with the BCR-ABL TKI imatinib [27].

Many B-ALLs carry translocations involving transcription factors [2], and genomic studies indicate that loss of histone acetyltransferases (HATs) is common [29, 30]. This suggests that targeting epigenetic factors, particularly histone deacetylases (HDAC), might have therapeutic benefit in B-ALL. The pan-HDAC inhibitor (HDACi) vorinostat is FDA-approved for cutaneous T cell lymphoma [31]. mTOR inhibitors have potential to suppress survival signaling, possibly enhancing the efficacy of HDACi. This concept is supported by preclinical studies of acute myeloid leukemia and hepatocellular carcinoma [32, 33], and is being tested in multiple clinical trials involving rapalogs combined with vorinostat or panobinostat (NCI clinical trial identifiers NCT00918333, NCT01341834, NCT01169532). A phase I trial of panobinostat and the rapalog everolimus reported 15% complete response rate in Hodgkins' lymphoma [34]. The effect of combining TOR-KIs and HDACi in B-ALL has not been reported.

We hypothesized that TOR-KIs might provide better efficacy than RAP when combined with HDACi in B-ALL. By blocking mTORC2, TOR-KIs reduce AKT activity and should increase the function of transcription factors in the Forkhead Box O (FOXO) family, leading to upregulation of pro-apoptotic genes [35]. We found that in human B-ALL cells, TOR-KIs are mainly cytostatic as single agents. Hence we tested whether HDACi could unleash the cytotoxic potential of TOR-KIs through epigenetic de-silencing of pro-apoptotic genes. The results show that HDACi increase the cytotoxic effects of TOR-KIs in B-ALL cell lines and primary B-ALL specimens. B-ALL cell killing by the HDACi/TOR-KI combination correlates with upregulation of FOXO target gene expression.

## RESULTS

### mTOR inhibition promotes cell cycle arrest but not death of B-ALL cells

**Figure 1:**
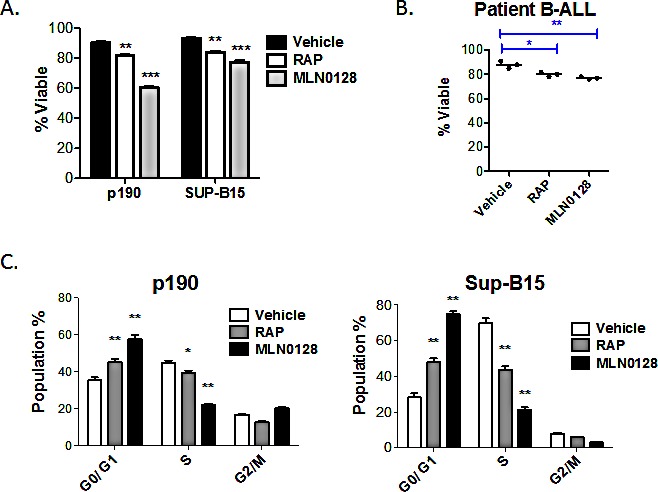
MLN0128 is mainly cytostatic in human B-ALL cells (A) Cell lines (p190, SUP-B15) or (B) primary B-ALL cells (n = 3 independent specimens) were cultured for 48hr with vehicle or with RAP or MLN0128. The percent viable cells was measured by 7-AAD staining and flow cytometry. For the primary patient samples, cells were grown on stromal cells and viability was determined for human CD19+ cells. (C) DNA content analysis was used to assess cell cycle distribution in p190 and SUP-B15 cells after 48 of culture. * p < 0.05; ** p < 0.01, *** p<0.001, one-way ANOVA.

**Figure 2:**
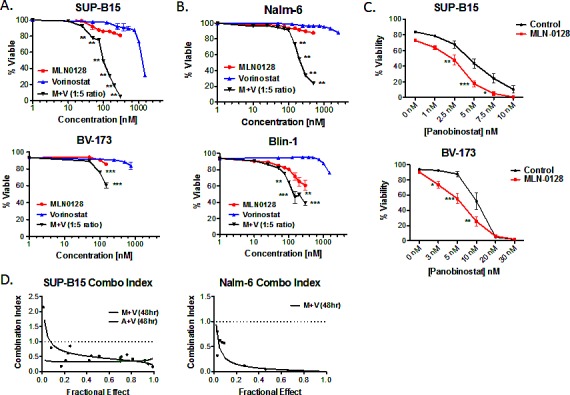
TOR-KIs and HDACi cause synergistic killing of B-ALL cell lines (A) Two Ph+ B-ALL cell lines (SUP-B15 and BV173) were cultured for 48hr with titrated concentrations of MLN0128, vorinostat or both. Viability was measured by 7-AAD staining. For the combination treatment, the values represent the concentration of MLN0128 for that condition; vorinostat was present at 5 times this concentration (for example, 100 nM MLN0128 and 500 nM vorinostat). * p < 0.05; ** p < 0.01, two-way ANOVA. (B) non-Ph B-ALL cell lines Nalm-6 and Blin-1 were analyzed as in panel A. (C) SUP-B15 and BV-173 cells were treated with the HDAC inhibitor panobinostat alone or in the presence of 100 nM MLN0128. (D) SUP-B15 and Nalm-6 cells were treated with combinations of TOR-KIs and vorinostat at fixed ratios for 48hr. Cell viability was determined, and the combination index for cell killing was calculated and graphed using Calcusyn software. The dashed line indicates a combination index of 1.

TOR-KIs provide significant anti-leukemic effects *in vitro* and *in vivo* using both murine and human models of B-ALL [27, 28, 36]. Consistent with our previous study using PP242 [27], the clinical candidate compound MLN0128 [28] caused both cell death (Fig. 1A) and G_0_/G_1_ arrest (Fig. 1C) in BCR-ABL-transformed murine pre-B cells (p190 cells). In contrast, human Ph+ cell lines (SUP-B15 and BV-173), Ph-negative cell lines (Nalm-6, Blin-1, RS11;4, 697, REH, SEM, Kasumi-2) and primary cells from bone marrow of pediatric B-ALL patients (Ph-negative) were less sensitive to MLN0128 induced cytotoxicity (Fig. 1A, 1B, 2A, 2B and Supplementary Figure S1). In agreement with our previous findings [27], TOR-KIs caused greater cell cycle arrest and death in p190 cells than rapamycin (Fig. 1A, C). Similarly, MLN0128 caused greater cell cycle arrest than rapamycin in SUP-B15 cells (Fig. 1C).

### HDAC inhibitors synergize with TOR-KIs to overcome B-ALL death resistance

Clinically relevant concentrations of the FDA-approved HDACi, vorinostat [37-42], did not affect the viability of a panel of Ph+ or non-Ph human B-ALL cell lines (Fig. 2A, 2B, S1). However, vorinostat significantly increased MLN0128-mediated cytotoxicity of Ph+ and non-Ph B-ALL cell lines (Fig. 2A, 2B and S1). Similar results were obtained using distinct combinations of TOR-KIs with pan-HDACi: AZD8055 with vorinostat (Fig. S2A), MLN0128 with panobinostat (Fig. 2C), or MLN0128 with Apicidin (data not shown). The combination of MLN0128 plus vorinostat caused significantly more death than rapamycin plus vorinostat (Fig. S2B), indicating an advantage of TOR-KIs relative to rapamycin.

**Figure 3:**
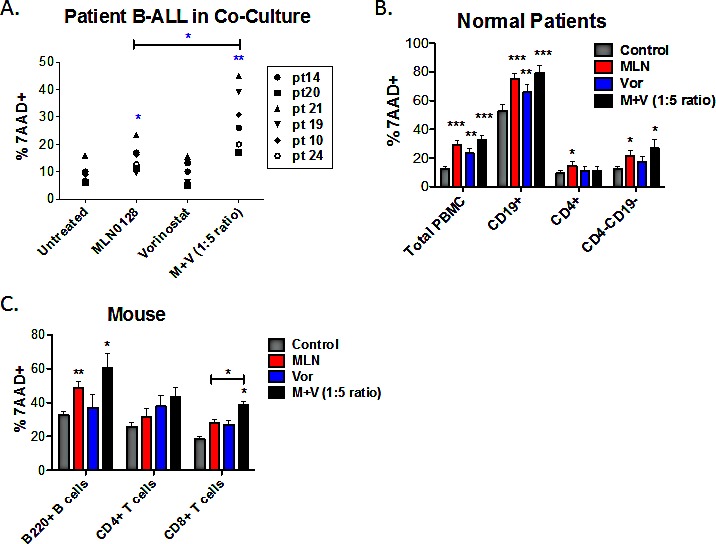
The combination of MLN0128/vorinostat increases killing of primary B-ALL cells with lesser effects on normal lymphocytes (A) Six non-Ph B-ALL patient specimens were cultured on stromal cells in the absence or presence of MLN0128 (100 nM), vorinostat (500 nM) or the combination. FACS was used to determine the percentage of hCD19+ cells that were non-viable (7-AAD-positive) after 48hr. (B) PBMCs from normal human donors were cultured for 48hr in media without cytokines. Cells were stained with anti-CD4 to mark helper T cells and anti-CD19 to mark B cells. Cytotoxicity was measured as the percent 7-AAD-positive as in Figure 3A. Viability of total PBMCs was determined using the ungated cells. Viability of CD4+, CD19+, and CD4-CD19- cells (which comprise mainly CD8+ T cells and natural killer cells) was determined using a lymphocyte gate based on forward and side scatter. Data represent mean +/− SEM (n = 5). (C) Purified mouse B-cells were cultured in BAFF and IL-4; purified T cells were cultured in IL-7 and IL-15. T cells were stained with anti-mouse CD4 and anti-mouse CD8 before viability analysis. Cytotoxicity was measured as the percent 7-AAD-positive as in panels A and B. For all panels, * p < 0.05; ** p < 0.01, unpaired two-tailed t-test.

The MLN0128/vorinostat combination showed a strong synergistic effect in the Ph+ cell line SUP-B15 (Fig. 2A) as well as the non-Ph cell line Nalm-6 (Fig. 2B). While the MLN0128/vorinostat combination enhanced cytotoxicity for all but one B-ALL cell line (REH, see Fig. S1) relative to single agent treatments, the magnitude of difference as well as inhibitor concentrations differed among the B-ALL cell lines. The heterogeneous response in cell lines prompted us to test the MLN0128/vorinostat combination on primary B-ALL cells. For these experiments, we maintained survival of pediatric B-ALL specimens by culturing on immortalized stromal cells as described previously [28]. MLN0128 alone caused a small increase in B-ALL death (Fig. 3A), consistent with the data in Fig. 1A. Vorinostat alone had no effect, but significantly enhanced B-ALL killing when added together with MLN0128 in each individual primary B-ALL specimen (Fig. 3A).

### Survival of normal lymphocytes treated with TOR-KIs plus HDACi

To evaluate the selectivity of the MLN0128/vorinostat combination for leukemia cells, we applied this drug combination to peripheral blood mononuclear cells (PBMCs) from normal human donors. After 48 hr of treatment, both MLN0128 (100 nM) and vorinostat (500 nM) slightly increased death of PBMC but the combination did not cause more death than MLN0128 alone (Fig. 3B). Gating on lymphocyte subpopulations showed that CD4+ T cells were largely resistant to MLN0128 or vorinostat alone or in combination (Fig. 3B). A significant but quantitatively small increase in killing was seen in the CD4-CD19- population (mostly CD8 T cells and natural killer cells) when treated with MLN0128 plus vorinostat. CD19+ B cells showed a high rate of spontaneous death following 48 hr *in vitro* culture, and this was further increased by MLN0128 (Fig. 3B). Titrating MLN0128 (10 – 750 nM) and vorinostat (50 – 3750 nM) confirmed greater effects on B cells than CD4+ T cells or CD4-CD19- cells (Fig. S3A, S3B).

Previously we reported that PP242 has minimal effects on survival and function of mouse T and B cells, when used at concentrations with anti-leukemic potential [27]. Similarly, MLN0128 at concentrations below 100 nM did not reduce survival of purified mouse splenic B cells cultured in the presence of cytokines IL-4 and BAFF (Fig. S3C). Vorinostat at concentrations below 1000 nM had minimal effect on mouse B cell survival (Fig. S3C). MLN0128 (100 nM) did increase death of mouse B cells but this was not increased further by vorinostat (500 nM) (Fig. 3C). The survival of mouse splenic T cells cultured in IL-7 and IL-15 was modestly reduced by treatment with MLN0128 and/or vorinostat (Fig. 3C, S3C). Overall these data suggest that MLN0128 and vorinostat, when combined at concentrations that cause B-ALL cell death, have little effect on human and mouse T cells but show cytotoxicity towards normal B cells.

### MLN0128/vorinostat induces apoptosis in B-ALL cells

**Figure 4:**
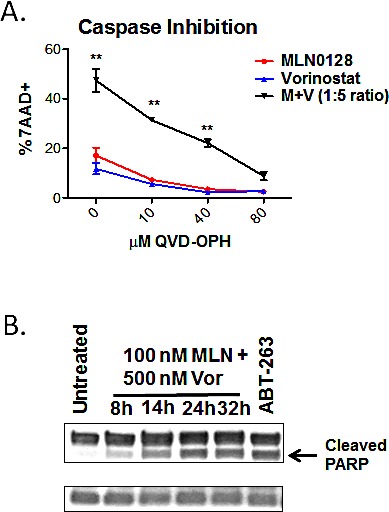
B-ALL cell death is caspase-dependent (A) SUP-B15 cells were cultured with MLN0128 (100 nM), vorinostat (500 nM), or the combination (M + V) in the presence of increasing concentrations of pan-caspase inhibitor. The percentage of cell death was determined after 48 hr by 7-AAD staining (mean +/− SEM, n = 3-5). * p < 0.05; ** p < 0.01, two-way ANOVA. (B) PARP cleavage in BV173 cells was measured by western blotting of lysates at various times after addition of MLN0128 (100 nM) and vorinostat (500 nM). Cells treated with the BCL2/BCL-XL antagonist ABT-263 were used as a positive control. β-actin served as a loading control. Similar results were observed in SUP-B15 cells.

B-ALL cell killing by combination treatment might induce cytotoxicity through necrosis or programmed cell death (i.e. apoptosis). The pan-caspase inhibitor QVD-OPH caused a concentration-dependent suppression of apoptosis in SUP-B15 cells treated with MLN0128/vorinostat (Fig. 4A), consistent with death by caspase-dependent apoptosis. The combination of MLN0128/vorinostat also increased PARP cleavage, a caspase-dependent event in apoptotic cells (Fig. 4B). We did not consistently detect cleaved caspases by immunoblot or flow cytometry (data not shown), perhaps owing to low levels of caspase expression in B-ALL cells [43, 44]. However, using enzyme assays we observed a transient increase in activity of both caspase-8 and caspase-3 in three B-ALL cell lines treated with MLN0128/vorinostat that was significantly higher than in cells treated with single agents (Fig. S4). Together these data support apoptosis as the cytotoxic mechanism of action for the MLN0128/vorinostat combination in B-ALL cells.

### mTOR inhibition promotes transcriptional activity of the tumor suppressor, FOXO

**Figure 5:**
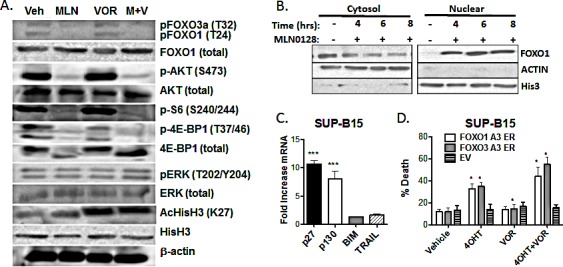
MLN0128 induces dephosphorylation and nuclear translocation of FOXO factors (A) Lysates from SUP-B15 cells treated for the indicated times with vehicle alone, MLN0128 (100 nM), vorinostat (500 nM), or the combination (M + V) Blots were probed for the proteins and phosphosites shown on the right. (B) SUP-B15 cells were treated for the indicated times with MLN0128 (100 nM) before cell fractionation into nuclear and cytoplasmic extracts. Fractions were subjected to western blotting with anti-FOXO1 antibody. Antibodies to β-actin and histone H3 were used to confirm the purity of cytoplasmic and nuclear fractions. (C) SUP-B15 cells were treated for 4 hr with vehicle or 100 nM MLN0128. mRNA was obtained and expression of the indicated gene products was determined by Q-PCR. Graph depicts the fold increase in mRNA in cells treated with MLN0128 versus vehicle (mean +/− SEM, n = 3). *** p < 0.001, one-way ANOVA. BIM and TRAIL mRNA was not increased at 6 or 8 hr post-treatment (not shown). (D) SUP-B15 cells were infected with retroviruses expressing a human CD4 marker gene lacking the cytoplasmic tail and magnetically sorted to enrich hCD4+ cells. The vectors contained no insert (EV) or cDNAs encoding triple-alanine mutants of FOXO1 or FOXO3a fused to the hormone binding domain of the estrogen receptor (ER). Cells were then treated with vehicle or 4OHT for 48hr in the absence or presence of vorinostat (500 nM). The percentage of cell death was determined after 48 hr by 7-AAD staining (mean +/− SEM, n = 3). * p < 0.05; paired two-tailed t-test.

Broad spectrum HDACi like vorinostat have the potential to alter cellular signal transduction through altered transcription or non-histone target proteins. To investigate the mechanism of synergistic killing of B-ALL cells by MLN0128/vorinostat, we first assessed phosphorylation of signaling proteins in the PI3K/AKT/mTOR pathway. As expected, vorinostat increased global histone acetylation in SUP-B15 cells; however, vorinostat did not affect phosphorylation of proteins in the PI3K/AKT/mTOR pathway (Fig. 5A). In contrast, MLN0128 treatment reduced phosphorylation of the mTORC1 substrate 4EBP1 (T37/46) and the mTORC2 substrate AKT (S473), with only a slight effect on histone acetylation (Fig. 5A). MLN0128 also blocked phosphorylation of ribosomal protein S6 (S240/244) downstream of mTORC1 (Fig. 5A). Similar results were observed in the Blin-1 and Nalm-6 cell lines (data not shown). MLN0128 and vorinostat did not influence ERK phosphorylation in SUP-B15 cells (Fig. 5A). These data suggest that vorinostat does not alter the activity of major oncogenic signaling pathways in B-ALL cell lines, nor does vorinostat modulate the signaling impact of TOR-KIs.

Next we considered possible changes in transcription factor activity. We noted that MLN0128 reduced phosphorylation of FOXO transcription factors in SUP-B15 cells (Fig. 5A). The FOXO family of transcription factors (FOXO1, FOXO3A, and FOXO4) function as redundant tumor suppressors in lymphocytes [45]. Activated AKT enters the nucleus and phosphorylates FOXO proteins, inhibiting their binding to DNA and promoting nuclear export [46]. Through this mechanism, PI3K/AKT signaling prevents FOXO-mediated transcription of target genes that promote cell cycle arrest and apoptosis [47, 48]. Of the known AKT substrates, phosphorylation of FOXO proteins is particularly sensitive to reduced mTORC2 activity and AKT-S473 phosphorylation [49, 50]. As we observed previously in PP242-treated p190 cells [27], MLN0128 promoted nuclear localization of FOXO1 in SUP-B15 cells (Fig. 5B). This led us to investigate further the potential role of FOXO in the cytotoxic effects of MLN0128/vorinostat.

First we assessed expression of FOXO target genes. Treatment of SUP-B15 cells with MLN0128 strongly upregulated mRNA expression of the cell cycle inhibitors p27 and p130, with lesser effects on the pro-apoptotic targets BIM or TRAIL (Fig. 5C). Next we tested the potential of FOXO factors to regulate cell death in SUP-B15 cells. Inducible activation of constitutively active FOXO factors (FOXO1-A3 or FOXO3A-A3) increased cell death by ~20% at 48hr, and by ~40% in combination with vorinostat (Fig. 5D). This indicates that forced activation of FOXO factors has a similar pro-death effect compared to TOR-KIs.

To obtain a broader view of gene expression changes following single or dual treatments, we used a PCR array to measure mRNAs for 90 genes regulating apoptosis. A heat map of the results shows that 8hr of treatment with vorinostat broadly increased expression of pro-apoptotic genes while reducing expression of pro-survival genes (Fig. S5). Combined treatment with vorinostat and MLN0128 also increased mRNA expression of a number of pro-apoptotic genes (Fig. S5). Several of these genes are known FOXO targets: *FASLG* (Fas ligand), *GADD45A*, *TNFSF10* (TRAIL), *TNFRSF10A* (death receptor-4 (DR4)), *TNFRSF10B*, (death receptor-5 (DR5)), *BNIP3L*. Of note, vorinostat alone altered expression of many genes to a similar or greater extent than the combination (Fig. S5). Therefore, it is likely that a complex reprogramming of gene expression drives cell death sensitivity in cells treated with TOR-KIs plus HDACi.

### Combining TOR-KIs and HDACi increases mitochondrial priming for death

**Figure 6:**
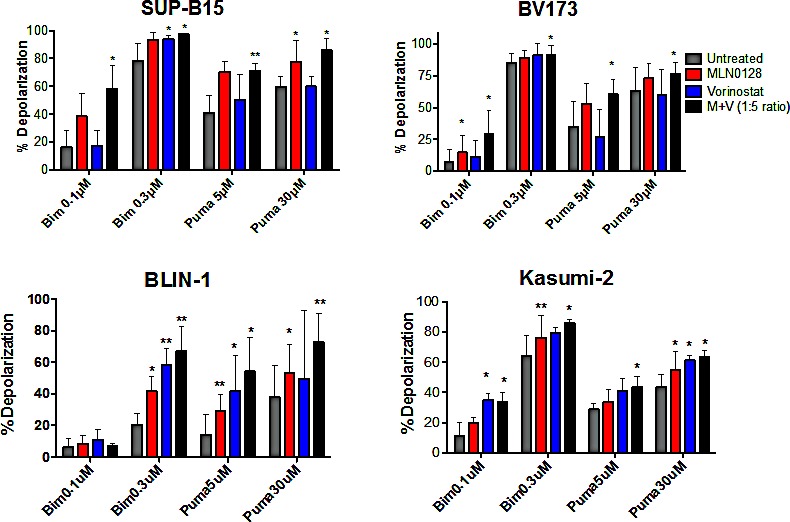
BH3 profiling assay shows increased mitochondrial priming in B-ALL cells treated with MLN0128/vorinostat Two Ph+ (SUP-B15 and BV173) and two non-Ph (BLIN-1 and Kasumi-2) B-ALL cell lines were subjected to BH3 profiling assay 16hr after treatment with vehicle (labeled untreated), MLN0128 (100 nM), vorinostat (500 nM), or the combination. Increase in mitochondrial depolarization is a quantitative measure of mitochondrial apoptosis initiation. In all lines, the combination increases the cells, sensitivity to pro-apoptotic BH3 peptides more than either agent alone. Percent depolarization of cells by each peptide is graphed. (mean +/− SD). n = 3-5; *p-value < 0.05; paired one-tailed t-test.

Due to the complex changes in gene transcription, we wanted to obtain a more direct understanding of how the drug combinations affect overall survival signaling at the mitochondria by using a BH3 profiling assay. The experimental approach involves treating permeabilized cells with fixed doses of death signals in the form of peptides made from the BH3 domain of pro-apoptotic BCL-2 family proteins, and measuring mitochondrial depolarization as in indicator of apoptosis initiation. An increase in the ratio of pro-apoptotic to anti-apoptotic factors at the mitochondria augments sensitivity to BH3 peptides and is termed mitochondrial “priming”. We found that 16hr of treatment with MLN0128/vorinostat significantly increased priming in four different B-ALL cell lines (Fig. 6). Interestingly, the cell lines varied in their response to individual drugs. These differences are best seen in the conditions where lower concentrations of Bim peptide (0.1 μM) or PUMA peptide (5 μM) were added. For example, vorinostat alone increased priming in Blin-1 and Kasumi but had little or no effect in BV-173 or SUP-B15 (Fig. 6). This variability, together with the PCR array data, suggests that the death mechanism triggered in B-ALL cells by MLN0128/vorinostat is complex and varies among cell lines. The identity of the pro-apoptotic genes upregulated by each inhibitor may vary depending on the particular epigenome and kinome of the cells. Regardless, in all cases the combination produced the greatest overall increase in priming, which correspondingly accounts for some of the synergy we observed in B-ALL cell killing.

## DISCUSSION

Inhibitors of the PI3K/AKT/mTOR pathway hold promise for treating cancer, and may be most effective when used in combination with other agents [25]. For example, rapalogs have demonstrated limited single agent activity in human cancer patients but provide significant clinical benefit in hormone-dependent breast cancer when given in combination with aromatase inhibitors [24]. We showed previously that combining TOR-KIs with tyrosine kinase inhibitors is effective in preclinical models of Ph+ B-ALL [27, 28]. Here we report that cytotoxic potential of TOR-KIs in B-ALL can be augmented by pan-HDAC inhibitors such as vorinostat or panobinostat. The synergistic effect is observed in Ph+ and non-Ph B-ALL cell lines as well as primary pediatric B-ALL.

Our data suggest that FOXO transcription factors may play a role in the death mechanism. FOXO proteins help to maintain quiescence in CML stem cells [51] and can promote apoptosis in CML cells treated with ABL tyrosine kinase inhibitors [52, 53]. TOR-KI treatment causes nuclear localization of FOXO proteins in B-ALL cells, shown here in SUP-B15 cells and previously in mouse p190 cells [27]. Moreover, we found that inducible expression of AKT-independent, constitutively active FOXO variants (FOXO1-A3, FOXO3a-A3) caused some cell death in SUP-B15 cells that was further increased by vorinostat, in the absence of TOR-KI treatment.

A plausible model is that HDACi treatment induces epigenetic changes that facilitate access of FOXO transcription factors to pro-apoptotic target genes in B-ALL cells. Results from the PCR array suggested broad reprogramming of gene expression by vorinostat alone or in combination with MLN0128. These changes result in B-ALL killing through activation of caspase-dependent apoptosis. The transient elevation of both caspase-8 and caspase-3 activity suggests that an extrinsic death pathway is involved. However, TRAIL was not required for B-ALL cell death in response to the MLN0128/vorinostat combination (data not shown). It is possible that multiple death receptors and ligands contribute to apoptosis.

The synergistic effect of TOR-KIs plus HDACi is greater than achieved using rapamycin plus HDACi. TOR-KIs are more effective inhibitors of mTORC2 but also suppress phosphorylation of rapamycin-resistant mTORC1 substrates, such as 4EBP1 [15, 22]. Hence, it is likely that the AKT-FOXO axis downstream of mTORC2 is not the only relevant target of TOR-KIs for achieving synergy with HDACi. By phosphorylating 4EBP1, mTORC1 promotes cap-dependent translation of mRNAs encoding pro-survival proteins (e.g. MCL-1) [8]. mTORC1 also controls transcription of metabolic genes to maintain cancer cell survival and proliferation [54]. The precise mechanism of synergy between TOR-KIs and HDACi might differ among B-ALL cell lines, depending on varying histone acetylation patterns. In support of this, the BH3 profiling assay showed that single agent treatment with MLN0128 or vorinostat had variable effects on mitochondrial priming among four cell lines tested. Previous reports of synergistic anti-cancer effects of HDACi with PI3K/mTOR pathway inhibitors have identified distinct mechanisms of tumor cell killing among different blood cancers [32, 55-58], further supporting the idea that the mechanisms of synergy are context-dependent.

Although vorinostat is an FDA-approved treatment for cutaneous T cell lymphoma, evidence is accumulating that drugs of this class have a limited therapeutic window especially in combination regimens. A clinical trial of panobinostat with everolimus in relapsed/refractory lymphoma resulted in some objective responses but a high rate of adverse events including thrombocytopenia [34]. A clinical trial of panobinostat monotherapy in Hodgkin's Lymphoma patients who relapse after transplant reported some efficacy but considerable hematotoxicity [59]. Our data show that the combination of MLN0128 plus vorinostat strongly reduces viability of B-ALL cells but also affects normal B-lymphocytes from humans and mice. Broad changes in the translational landscape may be a double-edged sword that increases B-ALL killing but also increases normal cell toxicity or suppresses cellular function. For example, vorinostat suppresses cellular immune responses mediated by human NK cells and T lymphocytes [60]. Refinements in drug selectivity might improve further the selectivity for leukemia cells. Agents targeting a single HDAC isoform or subgroup might maintain synergy with TOR-KIs in leukemia cells while preserving normal physiological cell functions. Overall our results show that the combination of TOR-KIs and HDACi can be a viable treatment for B-ALL, but the resulting broad changes in the transcriptional landscape may call for the use of more isoform-selective HDAC inhibitors to limit toxicity.

## MATERIALS AND METHODS

### Materials

MLN0128 was purchased from Active Biochem (Hong Kong), PP242 and AZD8055 from Chemdea (Ridgewood, NJ). Using cell-based assays we verified that MLN0128 and PP242 from these vendors had comparable activity as compounds provided under a Material Transfer Agreement by Intellikine (synthesized as previously described [28]). Vorinostat was purchased from LC laboratories, and panobinostat from Selleck. Antibodies and flow cytometry reagents were obtained from Cell Signaling, Invitrogen, eBioscience and Biolegend. TRAIL R2/DR5 agonist or soluble TRAIL antagonist antibodies were purchased from Enzo Life Science or R&D Systems.

### Cell Lines

Generation and propagation of pre-B p190 mouse cells have been previously described [27]. Nalm-6 and Blin-1 cell lines were kindly provided by Dr. David Rawlings (University of Washington), and the RS11;4, SEM, and 697 cell lines by Dr. Anthony Letai (Harvard Med. School). The Ph+ cell lines SUP-B15, Tom-1, BV-173 and non-Ph lines REH and Kasumi-2 were purchased from ATCC and DSMZ. Except for the p190 cells, all described cells represent human pre-B cell acute lymphoblastic leukemia (B-ALL). All cells were maintained at 37°C in 5% CO2, and RPMI1640 supplemented with 1% L-glutamine, 1% Pen/Strep, 0.5% HEPES, 0.1% beta-mercaptoethanol and 10% FBS.

### Mice

Balb/c and C57Bl/6 mice were kept in specific pathogen-free animal facilities at the University of California, Irvine, and procedures were approved by the Institutional Animal Care and Use Committee. We used 3-5 week old male and female Balb/c mice to obtain bone marrow for generation of p190 cell lines. 6-12 week old C57Bl/6 mice were used to isolate B and T cells from spleen and lymph node.

### Flow cytometry (FACS)

Surface phenotyping was performed and analyzed with methods previously described [27]. Data was acquired using FACSCaliber (Becton Dickinson) and analyzed with FlowJo software version 9.5.2.

### Normal mouse and human lymphocytes

Deidentified peripheral blood samples were obtained from normal donors. PBMCs were purified using a Ficoll gradient and 0.5 million cells per condition were treated with MLN0128 and/or vorinostat in 1 mL of media (RPMI1640 supplemented with 1% L-glutamine, 1% Pen/Strep, 0.5% HEPES, 0.1% beta-mercaptoethanol and 10% FBS). After 48 hours of treatment, cells were stained with hCD19-PE (Biolegend), hCD4-FITC (BD Bioscience), human TruStain FcX (Biolegend) and 7-AAD. Mouse splenic B cells were purified using EasySep Mouse B Cell Isolation Kit (Stem Cell Techology) from wild-type C57BL/6 mice. Cells were plated at 1 million per mL (RPMI1640 supplemented with 1% L-glutamine, 1% Pen/Strep, 0.5% HEPES, 0.1% beta-mercaptoethanol, 10% FBS, 60 ng/mL BAFF and 20 ng/nL IL-4) in 96 well plates at 100 μL final volume per condition. Cells were treated with MLN0128 and/or vorinostat for 48 hours and stained with B220-AlexaFluor488 (eBioscience), mouse TruStain FcX (Biolegend) and 7-AAD. Lymph nodes were harvested from C57BL/6 mice and plated at 2 million per mL in media supplemented with 10 ng/mL IL-7 and 10 ng/mL IL-15 in 48 well plate at 300 μL final volume per condition. Cells were treated with MLN0128 and/or vorinostat for 48 hours and stained with CD8-PE (eBioscience), CD4-FITC (eBioscience), mouse TruStain FcX (Biolegend) and 7-AAD.

### Primary leukemia samples and stromal co-cultures

We obtained bone marrow from newly diagnosed pediatric B-ALL patients at CHOC Children's Hospital under Institutional Review Board protocols approved by CHOC and by UC Irvine. Leukocytes were isolated from these pediatric specimens by centrifugation over Ficoll and stored frozen in aliquots. For stromal co-culture experiments, hTERT-immortalized human marrow stromal cell (MSC) (10^4^) (provided by D. Campana, St. Jude's Children's Research Hospital) were plated in 96 well plates in RPMI1640+10% FBS containing 1 μM hydrocortisone (Sigma). The following day, the media was replaced, and 10^5^ B-ALL cells were plated with hTERT-MSCs in AIM-V media (Life Technologies) with 10% FBS supplemented with human SCF, IL-3, IL-7, and FLT-3L (Peprotech) at 100 ng/ml. The co-cultures were immediately treated with indicated inhibitors/drugs. Forty-eight hrs post-treatment, cells were manually dislodged, stained with human CD19-FITC (Biolegend) and 7-AAD (Life Technologies) and immediately analyzed by flow cytometry.

### Western Blot and Nuclear Fractionation

Western blot and nuclear fractionations were performed as previously described [27, 61]. Except for anti-Actin (Sigma), all indicated antibodies were purchased from Cell Signaling.

### RNA Extraction, cDNA Synthesis and qPCR

Total RNA was isolated using the TRIZOL method (Invitrogen). To quantify transcript levels, equal amounts of cDNA were synthesized using the iScript cDNA synthesis kit (Bio-Rad) and mixed with the SYBR Advantage qPCR Premix (Clontech) and run on the iCycler iQ™ Real-Time PCR Detection system (BioRad). All primers for identified genes were designed using the open access PrimerBank software (pga.mgh.harvard.edu/primerbank). All samples were done in duplicate or triplicate. The 2^−ΔΔCt^ was used to determine fold change in mRNA levels relative to vehicle control samples (DMSO). *L32* was amplified as the internal control. The Apoptotic PCR Array (Qiagen) was performed as per manufacturers instructions.

### Cell Cycle and Cell Death

Cell cycle analysis was performed as previously described [27]. Briefly, cell cultures were washed with cell cycle buffer (PBS containing 5 mM EDTA) and subsequently fixed with ice-cold 70% EtOH dropwise while being vortexed (medium setting). Cells were fixed overnight at –20ºC. Samples were subsequently washed with cell cycle buffer and incubated with 2 mg ml–1 RNase A solution for 3 hr at RT. Cells were then stained with prodium iodide (0.5 mg/ml) for 1 hr at RT. The DNA fluorescence parameter (585/42 nm bandpass) using linear amplification was collected on a FACS Caliber equipped with a 488 nm laser line. Doublets and aggregates were excluded by pulse width versus area.

Cell death was analyzed for all indentified B-ALL cell lines and primary patient samples using 7-AAD/forward scatter (FSC) FACS analysis as previously described [62]. Gating of vehicle control samples (DMSO) was used to identify viable vs. non-viable cells. The results of 7-AAD/FSC analysis corresponded with PI/Annexin-V-FITC (Biolegend) when tested (data not shown). As previously shown by others, immunoblot analysis of cleaved caspase -9 or -3 in parental B-ALL cell lines such as SUP-B15, Nalm-6, REH-1, etc. is unreliable in detecting death by apoptosis [43, 44]. Therefore, we used the specific and highly sensitive [63-65] Luciferase Caspase-Glo^®^ -8 or -3/7 Assay Systems (Promega) to determine kinetic caspase-3/7 or -8 activation as per the manufacturers instructions. For caspase inhibitor studies, the pan-capsase inhibitor Q-VD-OPH (R&D Systems) was incubated at concentrations identified in the figure legend concomitantly with inhibitor/drug treatment and cell viability via 7-AAD/FSC was analyzed 48 hrs post-treatment.

### Inducible FOXO expression

Triple alanine mutants of FOXO1 and FOXO3A (FOXO1-A3, FOXO3A-A3) fused to the hormone-binding domain of the estrogen receptor (ER) were the gift of Dr. Boudewijn Burgering (University of Utrecht). The cDNAs were subcloned into the retroviral vector MSCV-IRES-hCD4 to drive bicistronic expression of the FOXO protein and a truncated human CD4 lacking the cytoplasmic tail. 293T cells were plated in IMDM medium and were ~80% confluent at time of transfection. Next 2.5 μg of the FOXO A3 ER or empty vector construct and 2.5 μg of pCL Ampho packaging vector were combined with XtremeGene HP DNA transfection reagent (Roche) before adding dropwise to 293T cells. The cells were incubated at 37°C and the medium replaced at 24 hrs. At 48 hrs, viral supernatant was collected and centrifuged to remove any 293T cells in suspension. For spinfection, SUP-B15 cells in log phase of growth were resuspended in the viral supernatant and 10 μg/mL of polybrene. The cells were centrifuged in a 6 well plate at 500 x g and 37°C for 2 hrs and incubated at 37ºC overnight. Viral supernatant was then replaced with regular growth medium and the cells rested for a day. The spinfection protocol was repeated a total of 3 times. Infected SUP-B15 cells were resuspended in MACS Buffer (PBS, 2mM EDTA, 0.5% BSA) and labeled with anti-hCD4 biotin antibody (1.0 μg/1 × 10^6^ cells; eBioscience) for 10 mins at 4oC. Labeled cells were pelleted, resuspended in MACS Buffer, and conjugated with anti-biotin microbeads (10 μL/1 × 10^7^ cells; Miltenyi). The cell suspension was loaded into a MS MACS separation column (Miltenyi Biotec) to purify only CD4+ SUP-B15 cells. To induce expression, purified SUP-B15 cells were plated at 4 × 10^5^ cells in 2 mL in a 12 well plate and treated with DMSO control, 200 nM 4-OHT, 500 nM Vorinostat, or 4-OHT+Vorinostat. After 48 hours, cells were labeled with anti-hCD4 Alexa 647 antibody (Biolegend) and 7-AAD. 7-AAD/FSC analysis was performed on hCD4+ cells using the BD FACSCaliber to detect cell viability.

### BH3 Profiling

B-ALL cell lines were incubated with no inhibitor, 100 nM MLN0128, 500 nM of vorinostat, or the combination for 16 hours. BH3 profiling was performed as described in Vo et al [66]. Briefly, cells were washed in PBS and resuspended at 2 x10^6^ per mL of (TEB) Trehalose Experimental Buffer (300 mM trehalose, 1 mM EDTA, 1 mM EGTA, 5 mM succinic acid, 0.1% BSA-IgG free, 10 mM HEPES, 80 mM KCl). Next 100 μL of cells was combined with 100 μL of TEB containing BH3 peptides (Bim or Puma), 20 ug/mL oligomycin, 0.005% digitonin. Then 25 μL of JC-1 (900 nM stock) was added. These components were incubated at room temperature for 90 minutes and red JC-1 (FL2 channel) aggregates were detected in each cell by FACS analysis immediately after.

### Statistical analysis

Two-way ANOVA was used with Tukey-Kramer *post-hoc* analysis for cell viability experiments. For BH3 profiling, a paired t-test was done to compare untreated versus each treatment group for each peptide to show how each treatment affects basal priming. We used GraphPad Prism (5.0c) software for all statistical analysis. To determine whether inhibitor combinations were additive, synergistic, or antagonistic, we used the Chou and Talalay median effects analysis to calculate a combination index (CI) using CalcuSyn v1 software (Biosoft) as described [67].

## SUPPLEMENTARY MATERIAL, FIGURES, TABLES


