# Supplementary material for: mTOR kinase inhibitors synergize with histone deacetylase inhibitors to kill B-cell acute lymphoblastic leukemia cells
Source: Oncotarget. 2014 Dec 12;6(4):2088–100. doi: 10.18632/oncotarget.2992 (PMC4385838; doi:10.18632/oncotarget.2992)
Supplement: Supplementary file 1 [file oncotarget-06-2088-s001.pdf]

mTOR kinase inhibitors synergize with histone deacetylase inhibitors to kill B-cell acute lymphoblastic leukemia cells

Supplementary Material

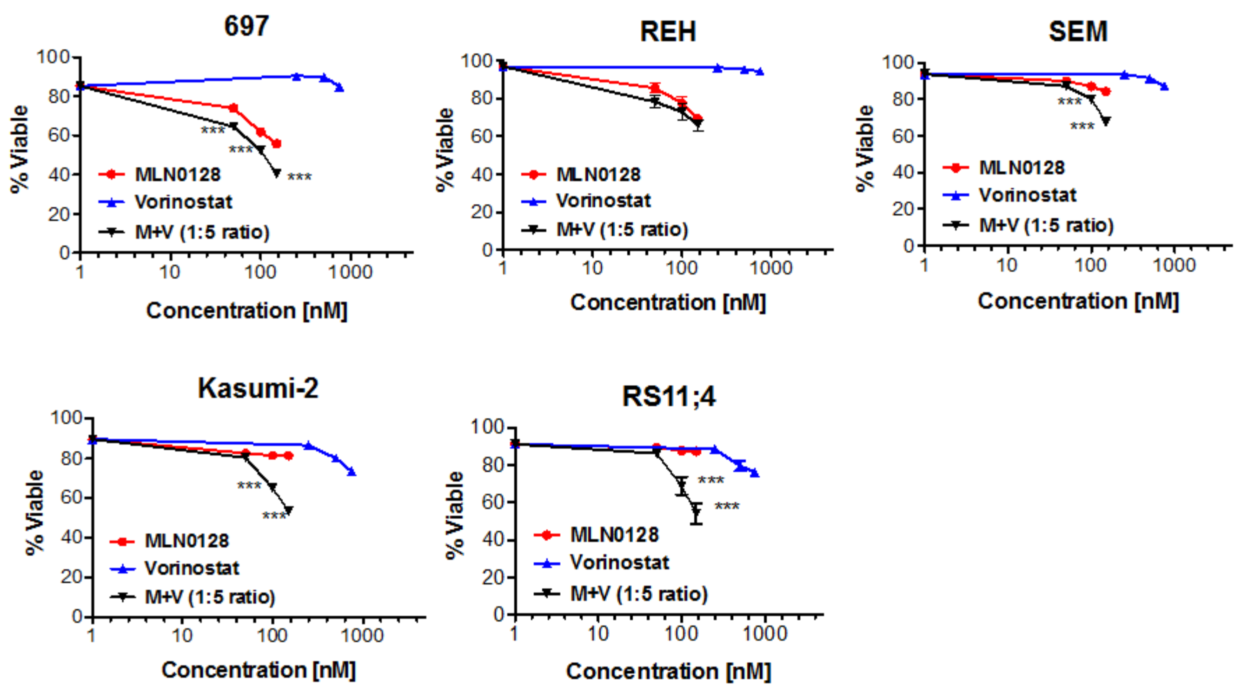

Fig. S1: Effects of single and dual treatments on a panel of B-ALL cell lines. Five non-Ph B-ALL cell lines were treated for 48hr with single or combination treatments. Viability was determined and graphed as in Figure 2A and 2B. *** $p < 0.001$, two-way ANOVA.

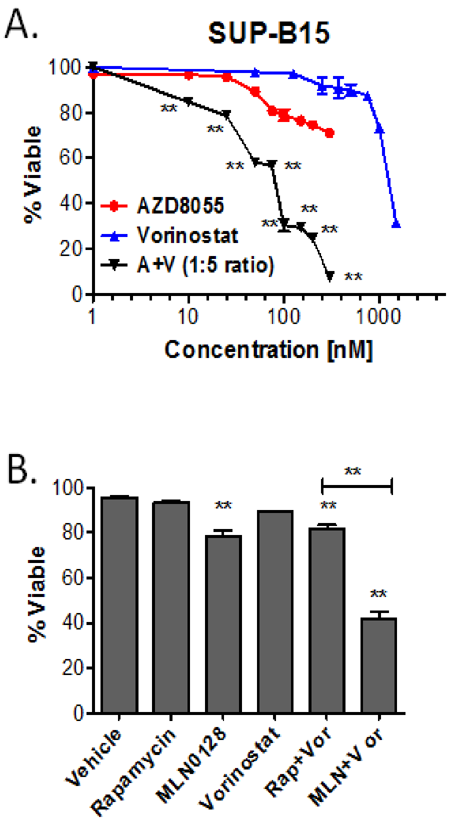

Fig. S2: Additional drug combinations. (A) The combination of AZD8055 plus vorinostat was tested in SUP-B15 cells. ** $p < 0.01$, two-way ANOVA. (B) MLN0128 plus vorinostat causes more death than RAP plus vorinostat. ** $p < 0.01$, one-way ANOVA.

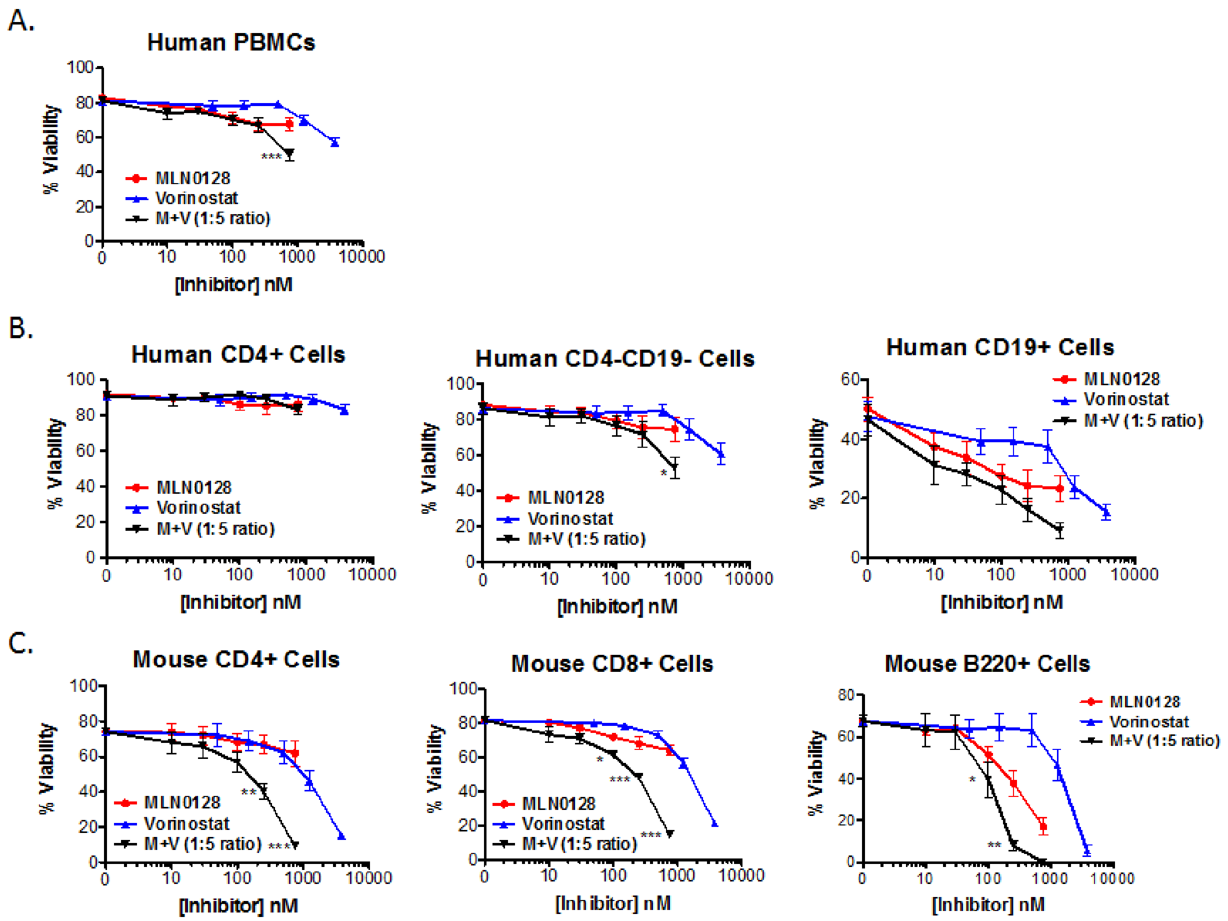

Fig. S3: Analysis of normal B and T cells. Viability of normal human (A, B) and mouse (C) lymphocytes was determined as in Figures 2A, 2B, S1 and S2A. Human PBMC (A, B) were cultured for 48hr in media alone without cytokines and titrated amounts of MLN0128, vorinostat or the combination at a 1:5 molar ratio. Viability in the total PBMC population or gated lymphocyte subsets was determined as in Figure 3B. n = 5. (C) Purified mouse B-cells were cultured in BAFF and IL-4; purified T cells were cultured in IL-7 and IL-15. Viability was determined as in Figure 3C with mean +/- SEM. n= 3* $p < 0.05$; ** $p < 0.01$; *** $p < 0.001$, two-way ANOVA.

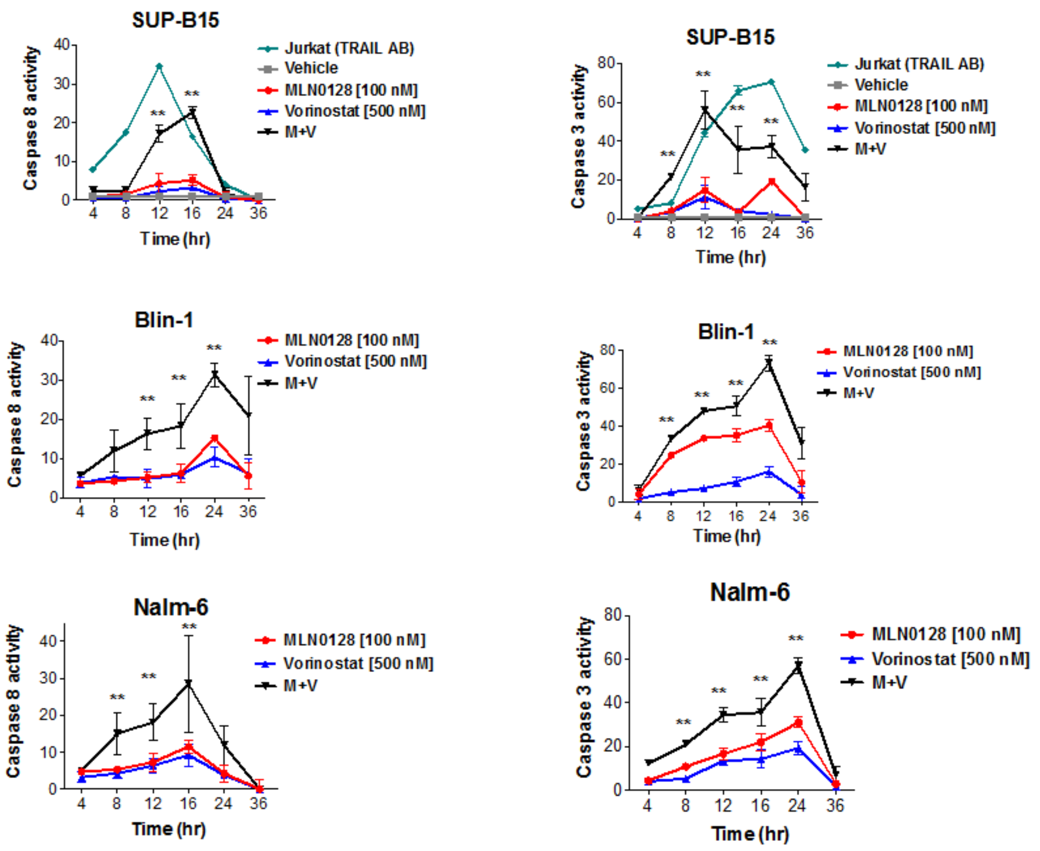

Fig. S4: Caspase enzyme assay data. Kinetic analysis of activated Caspase -8 or-3/7 via caspase-glo luciferase assay in SUP-B15, Blin-1 or Nalm-6 cells treated with vehicle (DMSO), 100nM MLN0128, 500nM vorinostat or corresponding doses of MLN0128 + vorinostat (M+V). Y-axis represents percent change in activated caspase for drug treated samples relative to vehicle sample for each time point (X-axis). (n= 3-5; ** $p < 0.01$ vs. both vehicle alone and MLN0128 alone, two-way ANOVA).

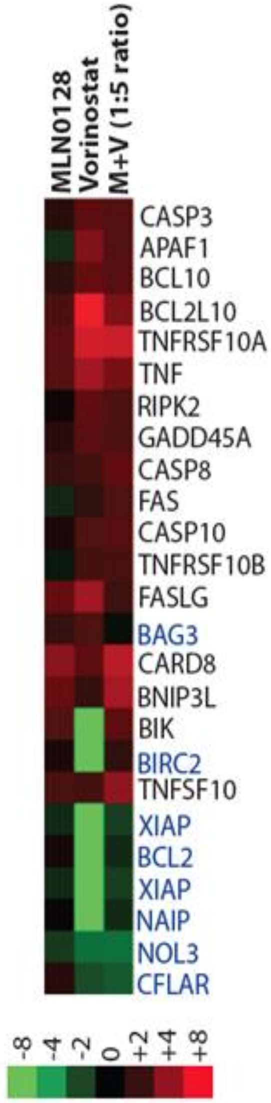

Supp. Figure 5: Vorinostat potentiates expression of pro-apoptotic genes including targets of FOXO factors. SUP-B15 cells were cultured for 8hr with vehicle alone, MLN0128 (100 nM), vorinostat (500 nM), or the combination (M + V). mRNA was extracted and used to determine expression of 96 gene products using a PCR array. Gene expression changes that passed array quality control checks were subjected to unsupervised hierarchical clustering based on spearman rank correlation (using cluster3 free software) and data presented as a heat map using Java

TreeView (free software). Pro-death genes are listed in black font and pro-survival genes in blue font.
